# Neural Mechanisms of Body Awareness in Infants

**DOI:** 10.1093/cercor/bhu261

**Published:** 2014-11-17

**Authors:** M. L. Filippetti, S. Lloyd-Fox, M. R. Longo, T. Farroni, M. H. Johnson

**Affiliations:** 1 Centre for Brain and Cognitive Development; 2 Department of Psychological Sciences, University of London, Birkbeck, UK; 3 Dipartimento di Psicologia dello Sviluppo e della Socializzazione, University of Padua, Padua, Italy

**Keywords:** body awareness, cognitive development, infant development, multisensory integration, near-infrared spectroscopy

## Abstract

The ability to differentiate one's body from others is a fundamental aspect of social perception and has been shown to involve the integration of sense modalities attributable to the self. Though behavioral studies in infancy have investigated infants' discrimination of body-related multisensory stimuli, whether they attribute this information as belonging to the self is still unknown. In human adults, neuroimaging studies have demonstrated the recruitment of a specific set of brain regions in response to body-related multisensory integration. To test whether the infant brain integrates this information similarly to adults, in a first functional near-infrared spectroscopy study we investigated the role of visual–proprioceptive feedback when temporal cues are manipulated by showing 5-month-old infants an online video of their own face while the infant was performing movements. To explore the role of body-related contingency further, in a second study we investigated whether cortical activation in response to self-initiated movements and external tactile stimulation was similar to that found in the first study. Our results indicate that infants' specialized cortical activation in response to body-related contingencies is similar to brain activation seen in response to body awareness in adults.

## Introduction

The construction and nature of the brain representations of one's own body have become an increasing focus of research in recent years. The use of paradigms such as the rubber hand illusion (RHI; Botvinick and Cohen 1998; Ehrsson et al. 2005; Tsakiris and Haggard 2005; Kalckert and Ehrsson 2012) and the enfacement illusion (Tsakiris 2008; Sforza et al. 2010; Tajadura-Jiménez, Grehl, Tsakiris 2012; Tajadura-Jiménez, Longo, et al. 2012) have shown that a rubber hand (RHI) or a morphed face (enfacement illusion) synchronously touched with our own hand or face can be perceived as belonging to ourselves (Botvinick and Cohen 1998; Tsakiris 2008). Such illusions suggest that multisensory integration is a key factor involved in producing awareness of one's own body. Nevertheless, despite increasing understanding of body awareness in adulthood, fundamental questions remain about the acquisition and development of body perception in infancy.

Research on body perception in infancy has shown: (1) that multisensory information is fundamental for the construction of body perception (Zmyj et al. 2011), and (2) that efferent signals are key factors for maintaining a coherent and unitary representation of our body (Bahrick and Watson 1985; Rochat and Morgan 1995; Schmuckler 1996; Morgan and Rochat 1997; Reddy et al. 2007). Furthermore, we have recently provided evidence of early body perception by demonstrating that human newborns can visually discriminate visual–tactile stimuli when the visual information is related to the body (Filippetti et al. 2013). Nevertheless, although investigating behavior in preverbal infants can provide evidence consistent with the infants' ability to attribute body-related multisensory information as belonging to the self, more direct converging evidence can potentially be obtained from neuroimaging. Studies on the neural bases of body awareness in adulthood have demonstrated the activation of a specific set of regions in response to body-related information (Tsakiris et al. 2008; Apps et al. 2013). Therefore, to establish whether similar neural mechanisms are also involved in body perception during infancy, we investigated the neural bases of body-related information in young infants.

Studies on the RHI with adults show that the right temporo-parietal junction (rTPJ) is involved in the processing of multisensory information attributable to the self (Tsakiris et al. 2008; Heinisch et al. 2011). A recent fMRI study using the enfacement illusion confirmed that the rTPJ is active in response to synchronous visual–tactile stimuli, as well as the involvement of the inferior occipital gyrus and the intra-parietal sulcus for self-identification (Apps et al. 2013). Furthermore, fMRI studies have confirmed the involvement of the right hemisphere in processing self-face recognition [for a review, see Devue and Brédart (2011)].

It has been suggested that multisensory regions of the cortex may have a key role in the induction of the RHI, when combined visual and tactile stimulation is experienced (Moseley et al. 2012). In fact, fMRI studies that have looked at perception of coherent and incoherent bodily movements have found specific activation over the superior temporal sulcus (STS) in response to biological motion (Leube 2003), with variation in activation as a function of the perceived level of discrepancy with one's own performed movements (Herrington et al. 2012).

Despite the lack of research on the neurobiological basis of body perception in infancy, the investigation of the social brain in infancy can provide indirect evidence of early perception of the self. An example emerges from a study by Lloyd-Fox, Blasi, Everdell, et al. (2011) that used functional near-infrared spectroscopy (fNIRS) to identify the brain regions involved in socially relevant, biological stimuli compared with non-biological mechanical movements. The authors found that the frontal and temporal cortices responded to biological movements, with different patterns of cortical activation in response to different body parts (i.e., eye, mouth, and hand). The authors speculated that the specific activation found in response to hand movements combined with direct gaze could be interpreted in relation to self-directedness and socially relevant ostensive signals (Lloyd-Fox, Blasi, Everdell, et al. 2011). Further support for these results emerges from recent work (Lloyd-Fox et al. 2013) that found a relationship between activation in the TPJ–STS region to these human action sequences and the degree of social responsiveness of the infants (as recorded from the Mullen Scales of Early Learning; Mullen 1995).

Here, we used fNIRS to investigate the brain regions involved in multisensory integration and contingency detection for the development of body awareness in young infants. fNIRS represents an ideal method for the investigation of cortical activation in the developing brain as it can be easily applied to infants and provides higher spatial resolution than EEG [for a review, see Lloyd-Fox et al. (2010); Gervain et al. (2011)]. While our investigation of body awareness in infancy was inspired by the adult literature, we have adapted the original paradigms of the RHI and enfacement illusion to be suitable for infants. In a first fNIRS study, we investigated the role of visual–proprioceptive feedback when temporal cues are manipulated by showing 5-month-old infants an online video of their own face while the infant was performing occasional movements. Crucially, the video display either matched or mismatched the infant's own bodily motion. To explore the role of combined visual–tactile and visual–proprioceptive information further, in a second study we investigated whether cortical activation in response to self-initiated movements and external tactile stimulation was similar to that found in the first study.

Our key hypothesis was that the perception of the contingent stimuli would result in activation over regions known to be involved in body awareness in adults, such as the TPJ and the STS (Tsakiris et al. 2008; Apps et al. 2013), and that additional multisensory stimulation during contingency will increase this activation.

## Materials and Methods

### Participants

Infants were recruited from a database of parents who had agreed to participate in child development studies.

Seventeen 5-month-old infants (9 girls, 8 boys; M = 151.9 days, SD = 10.6 days) took part in Experiment 1. Fifteen additional infants participated, but were excluded from further analysis due to fussiness (*n* = 9) or experimenter error (*n* = 2), failure to look at the minimum 3 trials per experimental condition (*n* = 2), or because the number of channels excluded due to motion artifact was higher than the allowed threshold (more than a third of the measurement channels; *n* = 2).

Eleven 5-month-old infants (5 girls, 6 boys; M = 151.4 days, SD = 8.7 days) took part in Experiment 2. Nineteen additional infants participated, but were excluded from further analysis due to fussiness (*n* = 4), experimenter error (*n* = 2), failure to look at the minimum 3 trials per experimental condition (*n* = 8), thick hair that prevented data collection (*n* = 2), or because the number of channels excluded due to motion artifact was higher than the allowed threshold (more than a third of the measurement channels; *n* = 3).

The local ethics committee approved the study protocol.

### Stimuli and Design

The design for both experiments comprised 2 experimental conditions and 1 baseline condition. The experimental stimuli consisted of online videos of the infants' face and upper part of the body. A video camera was placed just below the screen in a central position in order to film the infant's face and shoulders (therefore the infant could also see her upper arm movements). The infants were presented with videos of themselves from a specularly congruent position. Therefore, the occasional spontaneous movements of infants were always seen on the screen in a spatially congruent manner in both conditions.

The baseline stimuli consisted of full-color, static images of vehicles presented in random sequence, as used in a previous fNIRS experiment (Lloyd-Fox et al. 2013), and were identical in both experiments. These images were chosen to provide a non-dynamic and non-biological contrast to the body perception and a reference response from which to compare the activated period during the experimental conditions.

The session began with a baseline trial (12 s), followed by an experimental trial (15 s) and so forth, alternating one after the other. The 2 types of experimental trials were presented in an ABBA order (with the initial condition counterbalanced across infants) to prevent anticipatory effects. The experiment ended when the infants became bored or fussy as assessed by an experimenter who was monitoring their behavior.

In Experiment 1, the “contingent” condition presented the infant's face and upper part of the body in real time. As a result, the spontaneous movements of the infant were simultaneously displayed on the screen. In the “non-contingent” condition, the video presentation was delayed by 3 s (Watson 1994; Gergely and Watson 1999).

In Experiment 2, while the visual content of the 2 experimental conditions was displayed in an identical procedure to Experiment 1, visual–tactile stimulation was introduced together with visual–proprioceptive inputs. As a consequence, infants' were systematically stroked on the cheek with a soft paintbrush. However, whereas in the contingent condition both the visual–tactile stimulation and the visual feedback of the infant's executed movements were perfectly synchronous, during the non-contingent condition both seen and felt touch and movements were delayed. Stroking of the infant's cheek was manually delivered by the experimenter using a soft medium size paintbrush (width = 25 mm). Each stroke lasted for approximately 1 s; in order to ensure non-contingency between the seen and felt strokes, during the non-contingent condition the tactile stimulation was delayed with regard to the brush stroke displayed by 3 s (Watson 1994; Gergely and Watson 1999; Zmyj et al. 2009). A maximum amount of 3 strokes per trial were delivered in both conditions (M = 2.25, SD = 0.7 for the contingent condition; M = 1.69, SD = 0.6 for the non-contingent condition). The side of the stroke (right or left cheek) was counterbalanced across infants.

If necessary, occasional alerting sounds were played to draw the infant's attention back to the screen. To ensure that these sounds were balanced during the experimental session, each time the sound was used during the baseline trial, the following experimental trial would also include a sound.

### Procedure

Infants were tested in a dimly lit and sound attenuated room, and sat on an infant seat (Bebe Pod chair). The distance between the screen and the infant's head was approximately 90 cm. Infants were encouraged to watch the stimuli displayed on a 117-cm plasma monitor. Parents were asked to sit on a chair away from the infant's sight and to refrain from talking and interacting with the infant during the stimuli presentation unless the infant became fussy.

**Figure 1.**
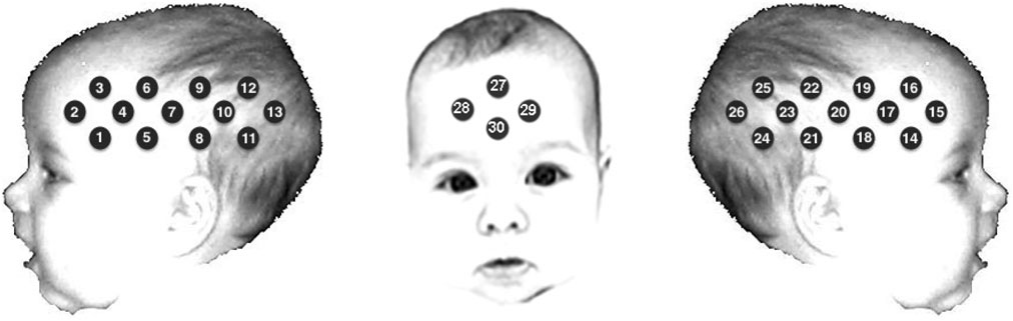
Schematic illustration of the fNIRS channel locations on an average 4- to 6-month-old head. The headgear of Experiment 2 comprised frontal channels (27–30), which were not included in Experiment 1. Note that the schematic is used for illustrative purposes only.

As illustrated in Figure 1, infants wore an in-house fNIRS headgear consisting of 3 source-detector arrays (Lloyd-Fox et al. 2013) and were tested using the UCL-fNIRS topography system (Everdell et al. 2005). This system used 2 continuous wavelengths of source light at 770 and 850 nm, and source-detector separations of 20 and 25 mm [for a review on the method, see Lloyd-Fox et al. (2010) and Gervain et al. (2011)]. While the headgear used with infants in Experiment 1 consisted of a total of 26 channels, we introduced prefrontal channels in Experiment 2, resulting in a 30-channel headgear (Fig. 1).

Before the study, measurements of the infant's head were taken to refer to external scalp landmarks and the 10–20 coordinates. For the group of infants in Experiment 1, the average head circumference was 42.72 cm (SD = 1.20), and the average distance from the glabella to the ear above the pre-auricular point (T3/T4) was 11.2 cm (SD = 0.69 cm). For the group of infants that took part in Experiment 2, the average head circumference was 42.96 cm (SD = 2.48) and the average distance from the glabella to the ear above the pre-auricular point (T3/T4) was 11.1 cm (SD = 0.66 cm). Therefore, the position of the channels over T3/T4 varied no more than 1 cm along the axial plane across infants, which allowed us to reliably individuate the location of cortical regions (Lloyd-Fox et al. 2014).

### Analysis and Data Processing

The looking behavior of each infant was coded off-line from a video recording of their eye movements. For a trial to be considered valid, the infant had to look to the screen for a minimum of 50% of the trial length. A minimum of 3 valid trials per experimental condition was required to include an infant in the fNIRS analysis. To maximize the likelihood that infants distinguished between the contingent and non-contingent conditions, trials where infants did not perform any movements (Experiment 1) or did not see the brush on the screen (Experiment 2) were excluded from further analysis. As a result of these exclusion criteria, valid fNIRS data were obtained from 17 infants in Experiment 1 and 11 infants in Experiment 2. This rate of attrition is slightly higher than in some previous fNIRS studies using the same headgear [see review from Lloyd-Fox et al. (2010)]. We believe this is due to the fact that the context was different from previous studies, as infants were asked to sit on a chair rather than on their parent's lap, sometimes leading to higher rates of fussiness and movement.

The recorded near-infrared attenuation measurements for each infant were initially analyzed, and trials and channels were rejected by looking time measures and the quality of the signals, using established artifact detection algorithms (Lloyd-Fox et al. 2010). Further analysis was performed with channels that survived these rejection criteria. Other inclusion criteria required: (1) that each channel contains valid data in both conditions, (2) a minimum number of 3 trials per condition, and (3) a maximum of 10 rejected channels (a third of the total measurement channels). The time window 10–18 s after the start of each experimental trial was selected to assess the degree of activation across infants. This period of time was selected to include the range of maximum concentration changes observed across infants for HbO_2_ and HHb. The maximum hemodynamic changes in both HbO_2_ and HHb concentration were analyzed. Either a significant increase in HbO_2_ concentration or a significant decrease in HHb is commonly accepted as indicators of cortical activation in infants (Lloyd-Fox et al. 2010). As an exclusion criteria, if HbO_2_ and HHb were to either increase or decrease significantly in unison, the signal was considered unreliable and excluded from the data set [for further discussion see Lloyd-Fox et al. (2010) and Gervain et al. (2011)].

The derived NIRS data were analyzed using standard paired sample channel-by-channel *t*-tests in which comparisons are performed between experimental conditions and between each experimental condition with the baseline (standard analysis; Lloyd-Fox et al. 2010). However, to control for false-positive channels, we also performed false discovery rate (FDR) analysis for both Experiments (FDR analysis; Singh and Dan 2006). The FDR is used in multichannel NIRS analysis to correct for multiple comparisons (Singh and Dan 2006).

Following the guidelines of Lloyd-Fox et al. (2014) on co-registered individual infant fNIRS–MRI data in 4–6 months old, we have used a standardized scalp surface map of fNIRS channel locators to identify our regions of interest within the frontal and temporal lobes. This map allows us to co-register the location of the response between our activated channels and underlying cortical areas.

## Results

### Experiment 1: fNIRS Results

**Figure 2.**
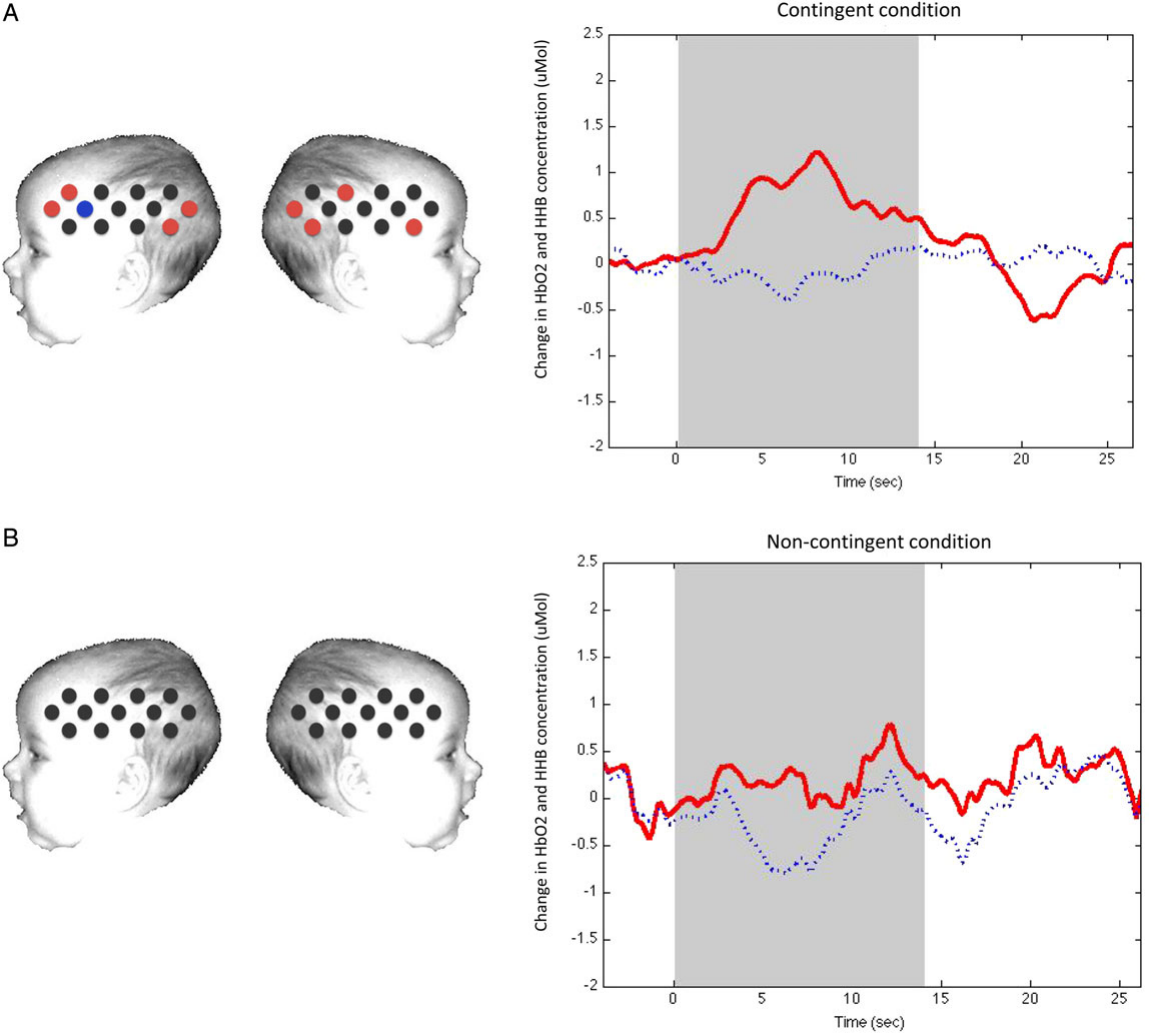
A schematic view of the fNIRS arrays with channels showing a significant increase in HbO_2_ during (*a*) the contingent condition compared with baseline and (*b*) the non-contingent condition compared with baseline (time window 10–18 s) in Experiment 1. Channels colored in red show a significant HbO_2_ increase in activation by standard analysis (not FDR corrected). Time courses on the right of the figure show HbO_2_ activation (in red) and HHb activation (in blue), in channel 26 during the contingent (top panel) and non-contingent (lower panel) conditions. Note that standard analysis is shown. No channels show a significantly greater hemodynamic change in the non-contingent condition compared with the contingent condition. Note that the schematic is used for illustrative purposes only.

Table 1 summarizes the significant hemodynamic responses to the contingent and non-contingent conditions relative to baseline in Experiment 1. The standard analysis of the response of the contingent stimuli relative to baseline revealed significant hemodynamic responses in HbO_2_ and HHb in 5 channels situated over a bilateral posterior region of the arrays and 3 channels in the anterior regions (Table 1 and Fig. 2). By using the standardized scalp surface fNIRS map (Lloyd-Fox et al. 2014), we identified the location of these posterior channels (channels 11, 13, 22, 24, and 26) as lying over the STS and TPJ cortical regions (these are defined by Lloyd-Fox et al. as the superior temporal-middle temporal gyri and superior temporal-postcentral gyri, respectively, as the atlas does not directly identify sulci; Lloyd-Fox et al. 2014), and the anterior channels (channels 2, 3, and 14) as over the inferior frontal gyrus (IFG) in both hemispheres.

**Table 1:** Results from the *t*-test “Channel-by-Channel Analysis” across the 2 experimental conditions in Experiment 1

Left lateral probe			Right lateral probe		
Channel	*P*-value	*t*-value	Channel	*P*-value	*t*-value
Contingent condition					
2	0.016	2.72	14	0.047	2.16
3	0.039	2.27	22	0.032	2.40
11	0.039	2.26	24	0.019	2.58
13	0.006	3.24	26	0.012	2.82
			**4**	**0.007**	**−3.07**
Non-contingent condition					
					

Note: Channels with significant activation are displayed for both conditions (*P* < 0.05). Channels that showed a significant decrease in HHb are in bold. In Experiment 1, the non-contingent condition did not reveal any channels with significant activation.

The standard analysis of the non-contingent condition revealed no significant HbO_2_ and HHb responses in any of the channels under investigation (Fig. 2). However, channels 14, 23, 24, and 25 showed a trend toward significance (in terms of HbO_2_ increase, *P* < 0.1).

Post hoc comparisons were made to control for false-positive activation using the FDR to correct for multiple comparisons (Singh and Dan 2006). As illustrated in Table 1, the application of the FDR led to the exclusion of all activated channels in the contingent condition. Considering the highly conservative property of the FDR for infant fNIRS analyses, we will refer to both analyses (standard and FDR) when discussing the results of the present experiments.

Paired sample channel-by-channel *t*-tests (two-tailed) were performed to compare responses with the contingent relative to the non-contingent condition (standard analysis). This analysis revealed a greater hemodynamic response to the contingent condition relative to the non-contingent condition in one channel located over the right posterior STS region (HbO_2_, channel 22: *t* = 2.92, *P* = 0.011, *d* = 0.76; Fig. 4).

### Experiment 2: fNIRS Results

**Figure 3.**
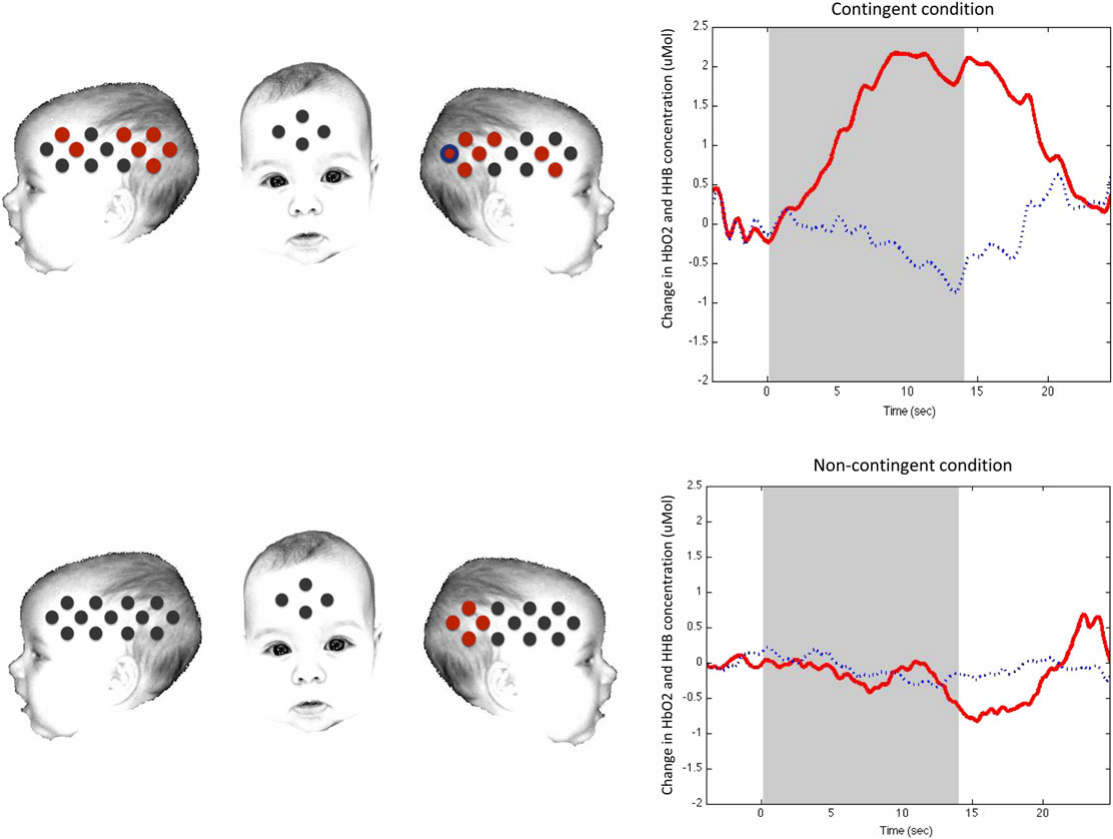
A schematic view of the fNIRS arrays with channels showing a significant increase in HbO_2_ during (*a*) the contingent condition compared with baseline and (*b*) the non-contingent condition compared with baseline (time window 10–18 s) in Experiment 2. Channels colored in red show a significant increase in HbO_2_, whereas channels in blue show a significant decrease in HHb concentration. The concomitant presence of the 2 colors highlights a simultaneous significant increase in HbO_2_ and a significant decrease in HHb. Time courses on the right of the figure show HbO_2_ activation (in red) and HHb activation (in blue), in channel 26 during the contingent (top panel) and non-contingent (lower panel) conditions. Note that standard analysis is shown. The schematic is used for illustrative purposes only.

Table 2 presents the significant hemodynamic responses to the contingent and non-contingent conditions relative to baseline in Experiment 2. The analysis of the response of the contingent stimuli relative to baseline revealed significant hemodynamic responses in HbO_2_ and HHb in 10 channels situated over a bilateral posterior region of the arrays and 4 channels in the anterior regions by standard analysis (Fig. 3). Channels activated in the contingent condition in the left and right hemisphere correspond to the STS and TPJ regions of the cortex, with 4 further channels active in the IFG in both hemispheres (Table 2 and Fig. 3).

**Table 2:** Results from the *t*-test “Channel-by-Channel Analysis” across the 2 experimental conditions in Experiment 2

Left lateral probe			Right lateral probe		
Channel	*P*-value	*t*-value	Channel	*P*-value	*t*-value
Contingent condition					
* 3*	*0.005*	*3.56*	*14*	*0.017*	*4.16*
* *4	0.032	2.49	*17*	*0.013*	*3.04*
* 9*	*0.013*	*3.03*	22	0.022	2.69
* 10*	*0.00005*	*6.73*	*23*	*0.003*	*3.93*
* 11*	*0.003*	*4.16*	*25*	*0.000004*	*8.97*
* 12*	*0.002*	*4.71*	*24*	*0.002*	*4.60*
* 13*	*0.0002*	*7.99*	*26*	*0.000009*	*8.25*
			**26**	**0.006**	**−3.49**
Non-contingent condition					
			23	0.03	2.61
			*24*	*0.0003*	*5.40*
			25	0.005	3.49
			26	0.039	2.37

Note: Channels with significant activation are displayed for both conditions (*P* < 0.05) (standard analysis). Channels that showed a significant decrease in HHb are in bold (standard analysis). Channels that survived after multiple comparisons correction (FDR) are displayed in italic.

The analysis of the non-contingent condition revealed significant HbO_2_ and HHb responses in 4 channels over the posterior region of the right array (Table 2 and Fig. 3), corresponding to the STS and TPJ regions (Lloyd-Fox et al. 2014).

Post hoc comparisons were made to control for false-positive activation using the FDR to correct for multiple comparisons (Singh and Dan 2006). As illustrated in Table 2, the FDR showed that, during the contingent condition, channels 3 (HbO_2_, *t* = 3.56, *P* = 0.005), 9 (HbO_2_, *t* = 3.03, *P* = 0.013,), 10 (HbO_2_, *t* = 6.73, *P* = 0.00005), 11 (HbO_2_, *t* = 4.16*, P* = 0.003), 12 (HbO_2_, *t* = 4.71, *P* = 0.002), 13 (HbO_2_, *t* = 7.99, *P* = 0.0002), 14 (HbO_2_, *t* = 4.16, *P* = 0.017), 17 (HbO_2_, *t* = 3.04, *P* = 0.013), 23 (HbO_2_, *t* = 3.93, *P* = 0.003), 24 (HbO_2_, *t* = 4.60, *P* = 0.002), 25 (HbO_2_, *t* = 8.97, *P* = 0.000004), and 26 (HbO_2_, *t* = 8.25, *P* = 0.000009) survived the multiple comparison analysis. The only channel of the non-contingent condition that survived the FDR is channel 24, which showed a significant HbO_2_ activation (*t* = 5.40, *P* = 0.0006). Overall, 5 channels did not survive the multiple comparison analysis.

**Figure 4.**
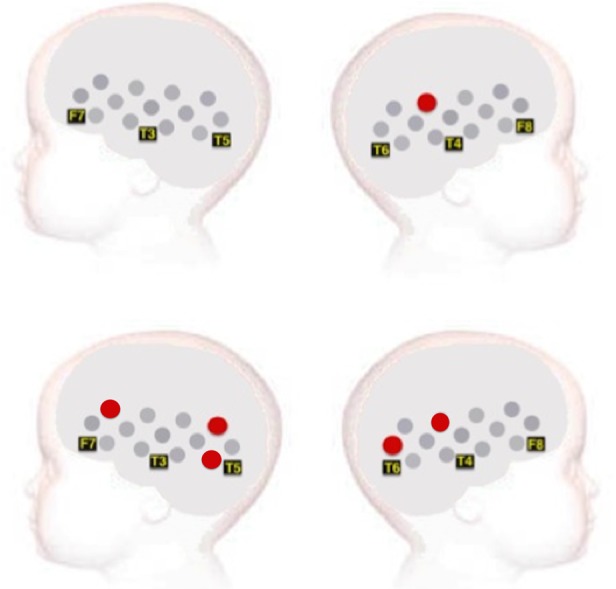
A schematic view of the sensor pads with channels showing a significant increased HbO_2_ activation during the contingent condition compared with the non-contingent condition and selected locations from the 10–20 arrays in Experiment 1 (top panel) and Experiment 2 (bottom panel). No channels show a hemodynamic change in the non-contingent condition compared with the contingent condition.

Paired sample channel-by-channel *t*-tests (two-tailed) were performed to compare responses with the contingent relative to the non-contingent condition. This analysis revealed a greater bilateral hemodynamic response to the contingent condition relative to the non-contingent condition in 4 channels located over the posterior STS region (HbO_2_, channel 11: *t* = 3.09, *P* = 0.015, *d* = 0.89; channel 12: *t* = 2.84, *P* = 0.025, *d* = 0.81; channel 22: t = 2.27; *P* = 0.047, *d* = 0.63; channel 26: *t* = 2.61; *P* = 0.026, *d* = 0.75) and 1 channel located over the left IFG (HbO_2_, channel 3: *t* = 2.55, *P* = 0.03, *d* = 0.99; standard analysis; Fig. 4).

### fNIRS Analysis Between Experiments

With the aim of determining the respective contribution of different types of multisensory information used in the 2 fNIRS experiments, contingency detection was compared across the 2 groups using a two-tailed independent-samples *t*-test in all channels, based on the standard analysis. We investigated the contingent > non-contingent differential response to measure the difference in response to the contingent condition between Experiments 1 and 2. Considering the small and different sample sizes of the 2 fNIRS experiments, we have applied the Welch *t*-test, which does not assume equal population variances.

**Figure 5.**
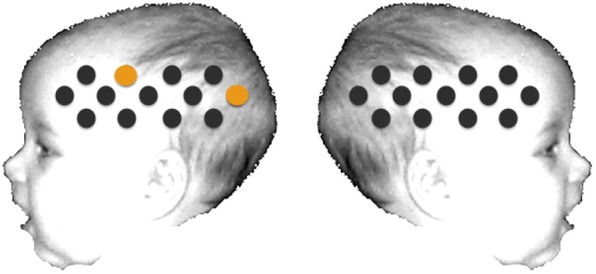
Analysis of the contingent response for Experiment 1 versus Experiment 2. The statistically significant effects (two-tailed, *P* = 0.05) are displayed for the contingent > non-contingent-selective responses. The channels that revealed a significantly greater response during the specified time window of activation are plotted in yellow and report an increase in HbO_2_ concentration.

As illustrated in Figure 5, in the left hemisphere this analysis revealed a significantly greater HbO_2_ contingent-selective response in Experiment 2 compared with Experiment 1 over 2 channels: channel 6 (*t* = 2.15, *P* = 0.04, *d* = 0.82) and channel 13 (*t* = 2.36, *P* = 0.03, *d* = 0.85). Additionally, in channel 14, positioned over the anterior right hemisphere, a trend toward contingency in the group of infants of Experiment 2 was found (*t* = 1.87, *P* = 0.07). In this age group, these channels are positioned approximately over the inferior frontal-precentral gyrus and STS region (Lloyd-Fox et al. 2014).

## Discussion

In this fNIRS study, we have identified areas of the infant cortex that specifically responded to contingent and non-contingent stimuli related to the body. To our knowledge, this is the first neuroimaging study to directly investigate the brain regions involved in body awareness in infancy. Overall, while in Experiment 2 we registered a widespread bilateral activation in response to visual–tactile and visual–proprioceptive contingency, the results of Experiment 1 showed weaker and relatively reduced cortical activation over similar areas of the brain. The weakness of the response in Experiment 1 was also confirmed by the lack of significant activation in the non-contingent condition (though the corresponding channels to those activated to the non-contingent condition in Experiment 2 did also show a trend toward significance in Experiment 1) and by the FDR correction.

By using a standardized scalp surface map of fNIRS channel locators to stable cortical regions within the frontal and temporal lobes (specific to 4–7 months old; Lloyd-Fox et al. 2014), we have been able to identify the most likely location of responses within the cortex. The activated areas of the cortex correspond approximately to the posterior STS and TPJ regions (which include regions covering the superior temporal-middle temporal gyri and superior temporal-postcentral gyri, respectively) and the IFG. The identification of the location of these responses allows us to compare the current findings with previous research with adults.

The adult literature shows that the STS region seems to be activated during the processing of social stimuli and biological motion (Leube 2003; Herrington et al. 2012). Furthermore, activation of the STS, right-middle and inferior temporal cortex has been associated with the perception of self-movements (Beauchamp et al. 2002), discrepancy between seen and observed movements (Leube 2003; Kontaris et al. 2009; Herrington et al. 2012), and affective touch (Bennett et al. 2013). For example, Leube (2003) ran an fMRI study with adults finding a positive correlation between the activation of right posterior STS and the temporal delay between one's own movements and its visual feedback. In this study, participants were asked to perform hand movements while watching their own action on a video screen, either online or delayed. Similar activation of this sub-region within the STS was reported by Herrington et al. (2012) in response to the incongruent perception of one's own movement, whereas coherent congruent motion activated the left STS. Furthermore, a recent study by Bennett et al. (2013) used fNIRS to investigate the brain regions activated by affective touch in adults, showing that the right posterior STS was activated in response to affective touch (arm) versus non-affective touch (palm).

In a recent fMRI study, Apps et al. (2013) found that the right inferior occipital gyrus, the right intra-parietal sulcus, and the right TPJ are involved in self-other overlap and self-identification during the enfacement illusion. Activity over these regions was modulated by synchronous and congruent visual–tactile stimulation between one's own and the other person face (Apps et al. 2013). Other adult studies that investigated the brain regions involved in body ownership have highlighted the role of the right TPJ in the distinction between self and other (Tsakiris et al. 2008; Heinisch et al. 2011), in visuo-spatial perspective taking, sense of agency, mental imagery of one's own body, and biological motion [for a review of these processes in out-of-body experience (OBEs) patients, see Blanke and Arzy (2005)]. OBEs following brain damage, or induced by intracranial stimulation of the TPJ, have been shown to involve, among other areas, the TPJ bilaterally (Blanke and Arzy 2005; Blanke and Thut 2007). This area seems therefore to be fundamental for the integration of visual–tactile signals with vestibular cues (Blanke and Arzy 2005). Our results are in line with these findings, showing the involvement of the temporal and parietal areas of the brain during body-related contingency.

The present 2 experiments also show cortical activation in the contingent condition over the IFG area. In an fMRI study with adults, Pelphrey et al. (2005) found activation, among other areas of the cortex, in the IFG in response to biological motion. Lloyd-Fox, Blasi, Everdell, et al. (2011) also found activation to a similar paradigm in infants using fNIRS, suggesting a specific role of the IFG in response to human actions such as manual movements and eye gaze shifts, which may trigger communicative intent. Because during the contingent condition of our fNIRS study infants were occasionally performing spontaneous movements with their arms and hands, it is possible that the visual–proprioceptive information as presented in the experiment may have triggered activation over the IFG. In contrast, because in the visual–proprioceptive mismatch information was not combined, these signals were not strong enough to trigger activation in the IFG (Lloyd-Fox, Blasi, Everdell, et al. 2011).

While results of Experiment 1 show a similar cortical response to the activation evidenced in response to combined visual–tactile and visual–proprioceptive information in Experiment 2, it is clear that the breath and strength of this response is considerably reduced in the first fNIRS experiment. In fact, we did not observe any significantly activated (above baseline) channel in response to the non-contingent condition. While on the one hand this lack of activation may emphasize the relevance of tactile experience in detecting contingencies, these findings may also highlight the presence of a caveat of the current experimental paradigm: because infants were not directly stimulated to perform self-movements, the chance that they could detect a match or mismatch of their action with its visual feedback was significantly reduced when compared with Experiment 2, where the amount of visual–tactile integration and disruption was controlled (Rochat and Morgan 1995). In the study by Rochat and Morgan (1995), infants were actively encouraged to move their legs (thus providing an interesting visual feedback on the screen). The authors used a sheet of paper and placed it under the infants' sit, together with a pin microphone which allowed to spread out the sounds that the infants made while kicking their feet. Potentially, this resulted in an increased amount of visual–proprioceptive feedback that the infant could use to make a distinction self-other. As a consequence, it might be that infants' cortical response to visual–proprioceptive contingency and non-contingency was weaker and less widespread, because the likelihood of detecting the difference between the 2 conditions was reduced. Indeed, the additional analysis that specifically investigated the role of movement in these 2 Experiments (Supplementary Material) seemed to suggest the importance of the amount of self-initiated movement for the detection of the contingent stimulation only in Experiment 8, where visual–proprioceptive information was provided. Therefore, one explanation is that the combination of 2 multisensory contingencies (namely visual–tactile and visual–proprioceptive) in Experiment 2 may have facilitated the detection of self-other differences. We speculate that visual–proprioceptive information may become more relevant when coordination and postural control are refined, making self-generated movements key information to perform a self/other differentiation. As a consequence, we expect that cortical activation seen in Experiment 1 would, later in development, mirror the hemodynamic response observed in Experiment 2 with 5-month-old infants.

Another caveat worthy of mention is related to the experimental design of these fNIRS studies. While we have increased chances of differentiation between the 2 conditions by excluding trials where the visual feedback was not detected, we cannot determine with absolute certainty whether the hemodynamic response reflected the elements of the behavioral epoch we were interested in. Future studies should investigate the topic by adopting an event-related design to establish the exact link between behavioral and cortical response.

Nevertheless, the present findings show that infants as young as 5 months of age have a specialized area of the temporal cortex for processing body-related information. This study is therefore the first to establish the presence of similar neural mechanisms of body perception between infancy and adulthood. In contrast to the previous work with adults, the current findings demonstrated a bilateral effect in the infants. Previous work on social action perception has reported bilateral effects (Lloyd-Fox et al. 2009), as well as the presence of reduced lateralization in infants compared with adults (Lloyd-Fox, Blasi, Mercure, et al. 2011). It is therefore possible that infants' response to body-related stimuli might initially engage more widespread activation than that seen in adults.

The analysis of the contingent > non-contingent-selective response in Experiment 2 compared with Experiment 1 revealed enhanced left lateralized activation to the combination of 2 multisensory contingencies compared with the visual–proprioceptive cues alone. This finding may suggest specialized cortical activation in response to combined visual–tactile/visual–proprioceptive information related to the body. Intriguingly, this result suggests that the combination of body-related cues in Experiment 2 may have maximized the multisensory stimulation attributable to the bodily self, providing crucial additional information for self–other differentiation in the infant brain.

It is possible that the differential response found in the contingent condition of Experiment 2 could also be explained in relation to the multisensory cues provided. STS and TPJ areas are known for their role in processing multisensory information (Beauchamp et al. 2008; Ionta et al. 2011; Noesselt et al. 2012). The present evidence of an enhanced response to the contingent visual–tactile and visual–proprioceptive cues may be interpreted in relation to the breadth of sensory stimulation provided. Nevertheless, the present findings suggest that infants process multisensory information related to the body and show a significantly reduced response when these stimuli are not presented in synchrony.

The current findings have important implications for our understanding of the development of body awareness. Crucially, our results suggest that common neural mechanisms support infant and adult processing of body-related stimuli. Previous studies that have investigated body perception in infancy have been unable to provide evidence that infants attribute body-related multisensory integration as belonging to the self. The present findings are the first to demonstrate that infants' specialized cortical activation in response to combined visual–tactile and visual–proprioceptive stimuli is similar to brain activation seen in response to body awareness in adults. Furthermore, our results also show that the infant brain responds similarly to visual–proprioceptive contingent cues alone, despite the overall cortical activation being weaker and less widespread. Overall, and in accord with the adult literature on body awareness (Tsakiris et al. 2008; Apps et al. 2013), we provide further evidence that multisensory integration and contingency detection is fundamental for body perception. Future research should investigate this topic further by exploring how different degrees of contingency (e.g., temporal and spatial invariants) are processed in the infant brain.

## Supplementary Material

Supplementary material can be found at: http://www.cercor.oxfordjournals.org/.

## Funding

This work was supported by the EC Marie Curie Initial Training Networks (MC-ITN-264301 TRACKDEV supporting M.L.F.) and the UK Medical Research Council (M.H.J.). T.F. is supported by the Wellcome Trust (073985/Z/03/Z) and the University of Padua. M.R.L. was supported by a grant from the European Research Council (ERC-2013-StG-336050) under the FP7. Funding to pay the Open Access publication charges for this article was provided by the UK Medical Research Council (reference number G0701484).

## Supplementary Material

Supplementary Data
