# Supplementary material for: Neural Mechanisms of Body Awareness in Infants
Source: Cereb Cortex. 2014 Nov 17;25(10):3779–87. doi: 10.1093/cercor/bhu261 (PMC4585515; doi:10.1093/cercor/bhu261)
Supplement: Supplementary Data [file supp_25_10_3779__index.html]

Neural Mechanisms of Body Awareness in Infants — Supplementary Data 

# Neural Mechanisms of Body Awareness in Infants

## Supplementary Data

Supplementary Data

**Files in this Data Supplement:**

- Supplementary Data - Docx file
