# Supplementary material for: Neural Mechanisms of Body Awareness in Infants
Source: Cereb Cortex. 2014 Nov 17;25(10):3779–87. doi: 10.1093/cercor/bhu261 (PMC4585515; doi:10.1093/cercor/bhu261)
Supplement: Supplementary Data [file supp_bhu261_bhu261supp.docx]

**Supplementary Material of**

Neural mechanisms of body awareness in infants

Filippetti, M.L.^1*^, Lloyd-Fox, S.^1^, Longo, M.R.^2^, Farroni, T.^3^, & Johnson, M.H^1^.

^1^Centre for Brain and Cognitive Development, Birkbeck, University of London, UK

^2^Department of Psychological Sciences, Birkbeck, University of London, UK

^3^ Dipartimento di Psicologia dello Sviluppo e della Socializzazione, University of Padua, Italy

*Correspondence: [m.filippetti@bbk.ac.uk](mailto:m.filippetti@bbk.ac.uk)

An additional analysis was performed in order to investigate the role of self-performed movement in body-related contingency detection. Both samples of the two fNIRS studies were divided into two groups: “high-movement” and “low-movement” infants. While infants in the “high-movement” group were infants who during the experimental session performed consistent movements in at least half of the trials in both conditions, infants comprising the “low-movement” group were those who didn’t meet this criterion. As a result, in Experiment 1, 9 infants were included in the “high-movement” group, whereas 8 infants comprised the “low-movement” group. In Experiment 2, the “high-movement” group included 4 infants, whereas the “low-movement” group comprised 7 infants.

**Results**

Figure 1S shows the cortical activation in response to the contingent condition vs. baseline in Experiment 1, for both groups of infants. While in Experiment 2 no significant difference in activation is registered between contingency vs. baseline in both groups (e.g. channel 23: “low-movement”, M = 1.42, SD = 0.55, “high-movement’, M = 1.14, SD = 1.56; channel 24: “low-movement”, M = 0.92, SD = 0.62, “high-movement’, M = 1.32, SD = 0.49; channel 25: “low-movement”, M = 1.49, SD= 0.84, “high-movement’, M = 1.60, SD = 1.32; channel 26: “low-movement”, M = 1.42, SD = 0.80, “high-movement’, M = 1.23, SD = 1.31), Experiment 1 shows that “high-movement” infants display a significant activation over STS and TPJ regions during contingency, as opposed to “low-movement” infants, which did not show any significant activated channel.


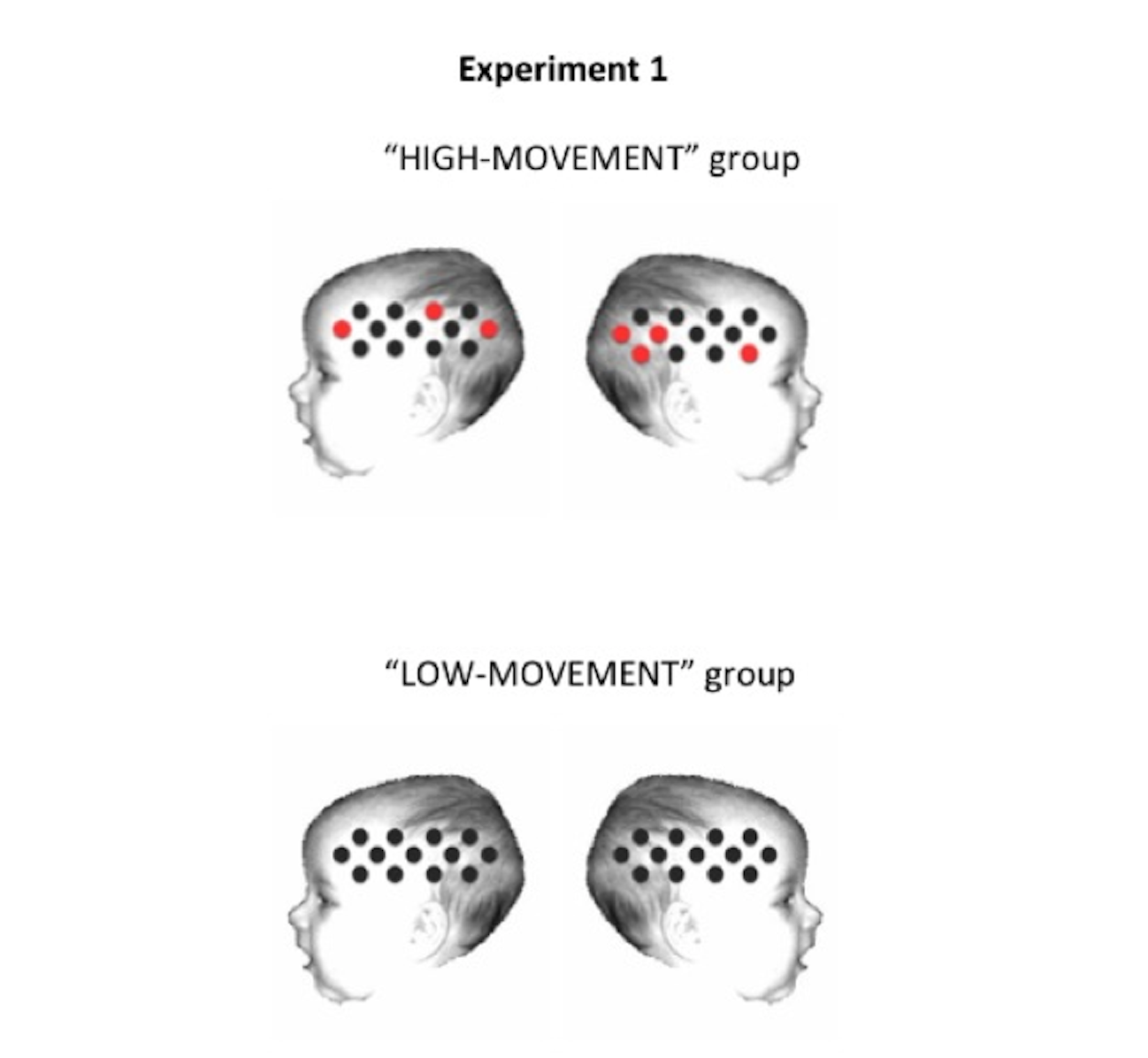


**Figure 1S**. A schematic view of the fNIRS arrays with channels showing a significant increase in HbO_2_ during the *contingent* condition compared to baseline in “high-movement” and “low-movement” infants in Experiments 1. A significant difference is present between infants included in the “high-movement” vs. “low-movement” groups.
